# The Existing Approaches to Sexuality Education Targeting Children: A Review Article

**Published:** 2017-07

**Authors:** Jila GANJI, Mohammad Hassan EMAMIAN, Raziyeh MAASOUMI, Afsanah KERAMAT, Effat MERGHATI KHOEI

**Affiliations:** 1. Dept. of Reproductive Health, Student Research Committee, School of Nursing and Midwifery, Shahroud University of Medical Sciences, Shahroud, Iran; 2. Center for Health Related Social and Behavioral Sciences Research, Shahroud University of Medical Sciences, Shahroud, Iran; 3. Dept. of Reproductive Health, Faculty of Nursing and Midwifery, Tehran University of Medical Sciences, Tehran, Iran; 4. Dept. of Reproductive Health, School of Nursing and Midwifery, Shahroud University of Medical Sciences, Shahroud, Iran; 5. The Iranian National Center for Addiction Studies (INCAS), Tehran University of Medical Sciences, Tehran, Iran; 6. Family & Sexual Health Division, Brian & Spinal Cord Injury Research Center (BASIR), Institution of Neuroscience, Tehran University of Medical Sciences, Tehran, Iran

**Keywords:** Sexuality education, Children, Parents, Review

## Abstract

**Background:**

We aimed to assess what is already known about sexuality education (SE)-related policy or practical issues using review methods to search and critically appraise the existing SE approaches targeting children under age 12 yr.

**Methods:**

We completed the data collection by an extensive search of the English and Persian published and unpublished literature, evidence from experts in the topic, and by searching citations. The MeSH-terms were sexuality and training, sexuality education and programs or approaches, sexuality and children, sexuality education and parents, sex or sexuality education, sex education and parents or caregivers. A systematic search of medical and health-related databases, the Cochrane Library and Web of Science was undertaken for the years 1970–2015 together with citation searching, reference list checking and recommendations from stakeholders to identify evidence for SE.

**Results:**

According to the inclusion criteria, 20 documents were identified. They were synthesized into three main categories as sexuality-related knowledge, attitudes, and parents’ skills to manage children’s sexual behavior and related education. Employed approaches to children’s sexuality were reported to be effective in developing healthy sexual behavior in children. Education was identified as the primary focus of the included packages and guidelines. Parents were recognized as first line educators in SE. However, interventions aiming to improve parents’ skills in SE for children were limited. In other words, developing skills in parents, and their competency in children’s sexual behavior management were not specified in the existing programs.

**Conclusion:**

Parents’ skill-building must be the focus of SE programs in order to address children’ sexual development goals.

## Introduction

Sexuality has physical, social, cultural and psychological dimensions and sexual development is part of human being’s life. This dimension, as the other aspects of human development, begins at birth reflected in one’s sexual behaviors (1). Sexual behavior is the result of a deeper and more complex process called “sexual socialization” (2–5). In other words, sexual behaviors are not only influenced by biological factors, but they also become complicated through sexual socialization. Children’s sexual behaviors are strongly influenced by children’s age and by how they have been socialized. Children’s sexual socialization is affected by the family and society’s belief and their function with respect to sexual matters (6–9).

Sexual socialization is a process through which children acquire the essential beliefs, attitudes, values, cultural symbols, concepts and meanings on sexuality (10, 11). In fact, identity formation, role of sex, sexual skills and knowledge acquisition, and development of sexual attitudes are achieved in this process (12). Family, as the first social group those children belong to from the early years of their lives; is considered the first and the most important factor effective in children’s sexual socialization (10, 11). Children acquire their knowledge, skills, and behavior from home, school and society, and the skills they gain can change their future (13). Therefore, SE by parents, as one of the main components of sexual socialization, is one of the best strategies for children’s sexual health promotion (14, 15).

Sexual behaviors are common in children and more than 50% of children get involved in different types of sexual behaviors before the age of 13 (16). Like other age groups, children need good care, supervision, and education during their sexual development, and their main caregivers are their first line educators (17, 18).

Parents are children’s first and foremost teachers in the field of sexuality. Most parents have not received such education and when it comes to SE, they tend to assign to schools what they themselves are not willing to do (15).

In Iran, the majority of parents are not well educated with regard to sexuality-related issues. In addition, there is no school-based sexual health education (19). As a result, it’s hard and fearful for parents to engage their children in conversations about sexuality (18).

Despite the importance of parents’ role in SE, they are not adequately prepared to communicate about sexual issues (20–23). They are mainly unable to manage properly their children’s sexual behaviors. Parents lack the adequate skills in empowering their young children to protect themselves against sexuality-related risks (24), enjoy sex in adulthood, and get prepared for a healthy and intimate interpersonal interaction (25, 26).

Using comprehensive programs and appropriate strategies for educating children on sexuality seems to be essential (27, 28). Cultural influences may alter the efficiency of any educational programs (29, 30). This review aimed to assess what is already known about SE-related policy or practice issue, by using systematic review methods to search and critically appraise the existing SE approaches targeting children under age 12.

## Methods

In order to complete this review within a very short time-frame, rapid review methods were used to ensure the efficient identification and synthesis of the most relevant evidence. The following keywords were used for search: sexuality training, sexuality education, sex education, sexual health, skill building, guidelines, packages, and children. Medical Subject Headings (MeSH) were also used. The terms included sexuality education and program or approach, sexuality and children, sexuality education and parents, sex or sexuality education, sex education and parents or caregivers. A systematic search of medical and health-related databases MEDLINE, EMBASE, PubMed, Cumulative Index to Nursing and Allied Health Literature (CINAHL), The Cochrane Library and Web of Science, Scopus, Google scholar, SID, Magiran, and Iranmedex was undertaken for the years 1970–2015 together with citation searching, reference list checking and recommendations from stakeholders to identify evidence for SE. The rationale for limiting the review to 1970–2015 was that sex education for children was originated from the Western societies. Many American kindergartens started to implement sexuality education curriculum since 1960s, and Sweden implemented sex education for all children and adolescents since 1970 (25).

We also searched key organizations and associations including WHO, UNICEF UNAIDS and Ministry of Health in countries such as Canada, Australia, the U.S., and Iran as well as active associations in the field of sexual health for children. In cases where the reported results were incomplete, the authors were contacted and asked for further details. The articles and gray documents were assessed based on the inclusion and exclusion criteria.

Inclusion criteria were studied design (articles, gray documents, packages and guidelines introduced in the field of SE); outcomes; and population (children aged 0–12); and interventions (designed to improve child sexual development through the provision of relevant knowledge, attitude, and skills of parents). Studies published in English and Persian was included in the study. We excluded programs targeted at the adolescent and the elderly. Duplicate publications of the same study and articles available only in abstract form were also excluded. Studies that met the inclusion criteria were critically appraised to assess their quality. Guideline Evaluation Tool (38) was used to assess study quality. This tool assesses concepts and topics covered (human development, relationships, personal skills, sexual behavior, sexual health, society and culture), accuracy and relevance (information is scientifically accurate; information is up-to-date; information is presented in a way that appeals to young people; information, graphics, and materials represent target populations).

Two reviewers separately screened the search results for inclusion using a predefined inclusion criteria form. The guidelines and packages contents were evaluated based on their applications in improving parents’ knowledge, attitudes and skills in SE and sexual behavior management for children under 12 yr of age.

Ethics Committee of Shahroud University of Medical Sciences approved this review with the ethical code of IR.SHMU.REC.2015.48.

## Results

**Fig. 1:**
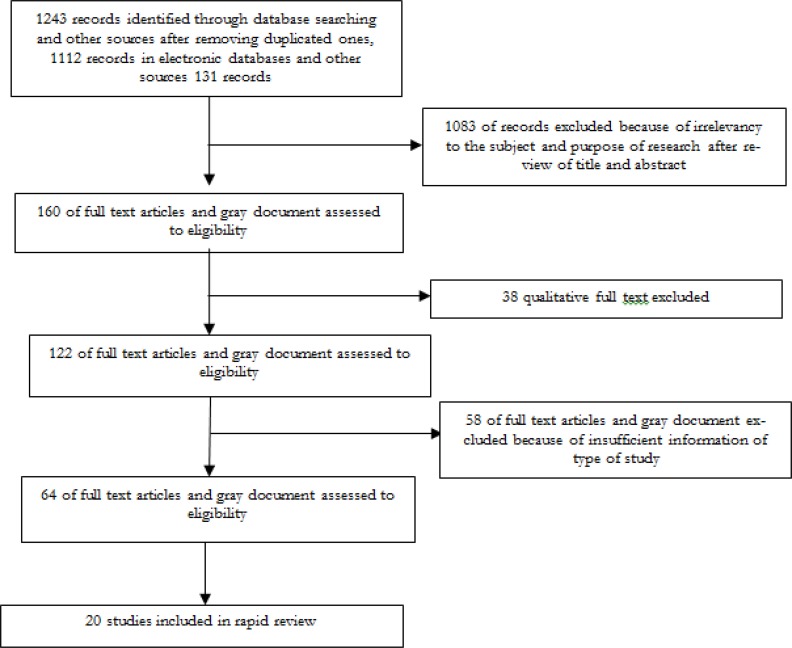
Flow diagram the study selection process for review

Of 1243 studies initially identified, after some exclusion, 20 studies from different regions of the world were included in our as study shown in (Fig. 1). The packages and guidelines were classified into three main categories based on sexuality-related knowledge, attitude, and skill (Table 1). Employed approaches to children’s sexuality were reported to be effective in developing healthy sexual behavior in children. Education was identified as the primary focus of the included packages and guidelines. Parents were recognized as first line educators in SEs. However, approaches regarding improving parents’ skills in SE for children were limited in number. In other words, skill-building approaches targeting parents, and parents’ competency in children’s sexual behavior management were not specified in the existing programs.

- Knowledge: provides accurate information about human sexuality, including growth and development, reproductive system, normal sexual behavior, childbirth.
- Attitude: offers opportunities for identification of values, beliefs and culture (personal, family, friends, and community).
- Skill: promotes the acquisition of skills in relation to competency in children’s sexual behavior management, impact on children’s moral growth and development, the ability to make healthy decisions, self-confidence, and sense of comfort with oneself and one’s body, understanding of children’s normal sexual behaviors, appropriate response to children’s sexual questions, identification and reporting of child sexual abuse.

**Table 1:** Characteristics of packages, guidelines and strategies available in sex education

**Number**	**Title**	**year**	**Organization**	**Country**	**Target group**	**Classification**
1	Talking to your preschool children about sexuality Parent Package 6 yr old and younger(31)	2009	Albert a Health Services	Canada	Parents	Knowledge, Attitude, Skill
2	Talk soon. Talk of ten. A guide for parents talking to their kids about Sex (32)	2011	Government of western Australia of Department of health.	Australia	Parents	Knowledge, Attitude, Skill
3	Understanding the Sexual behaviors of young children A Guide for parents and professionals(33)	2007 Sep.	Fair fax country Department of Family Services child help USA/Virginia.	USA	parents and professionals	Knowledge, Attitude, Skill
4	Talking to your children about Sexuality Parent Package 7–12 yr old(34)	2013	Alberta health services	Canada	parents	Knowledge, Attitude, Skill
5	Sexual Development and behavior in children information for parents and caregivers(35)	April 2009	The National Child traumatic Stress Network.	USA	parents	Knowledge, Attitude, Skill
6	International Technical Guidance of Sexuality Volume II(36)	2009 Dec.	United Nations Educational, Scientific and cultural organization (Unesco)	-	teachers and health educators	Knowledge, Attitude, Skill
7	Sexual Assault awareness. It’s time to talk it! Talk early, talk of ten. Parent sexual a violence(1)	2013	National Sexual Violence Resource Center	Canada	Parents and educators and professionals	Knowledge, Attitude
8	Children’s sexual behaviors a parent’s guide(37)	2013	The Provincial Child Sexual abuse advisory committee. Government of Prince Edward Island	Canada	Parents	Knowledge, Attitude, Skill
9	Guide lines for comprehensive sexuality Education 3^RD^ editor kindergarten through 12^th^ Grade(38)	2004	National Guide lines task force Sexuality information and education council of the united states (SIECUS).	U.S.A	teacher	Knowledge, Attitude, Skill
10	There’s No place like home sex education parent Guide(39)	2015	Mary Gossart. Planned parenthood health services of southwestern Oregon.	U.S.A	teachers and educators and parent	Knowledge, Attitude, Skill
11	Sexual development in primary aged children(40)	2007	Devon County Council ROYAL Devon and Exeter NHS. NHS Foundation trust.	Britannia	teachers	Knowledge, Attitude
12	Children’s sexual Behavior and body safety a Guide for Parents(41)	-	The children’s Assessment center in Grand Rapids, Michigan	U.S state of Michigan	parents	Knowledge, Attitude, Skill
13	Who Regional Office For Europe and BZgA Standards For Sexuality Education in European(42)	2010	Federal center for health Education, BZgA cologne.	Germany	policymakers, educational and health authorities and specialists	Knowledge, Attitude, Skill
14	Canadian Guide lines for sexual Health Education(43)	2003	Published by the authority of the Minister of Health Community Acquired Infections Division Center for Infectious Disease Prevention and Control, Health Canada, KIA OK9.	Canada	parents	Knowledge, Attitude
15	Parents approaches to educating their pre-adolescent and adolescent children about sexuality(44)	2009	University college Dublin and Queen’s university, Belfast	Northern Ireland	parents	Attitude
16	Keeping kids .safe A Guide for parents and caregivers(45)	2005	Tennessee Department of Human Services.	Tennessee State in USA	Parents and Caregivers	Knowledge, Attitude, Skill
17	Raising Healthy kids Families talk about sexual Health For parents of young children(46)	2003	Family Health production, Inc.	USA	parents	Knowledge, Attitude, Skill
18	What to say when they ask Talking about sexuality with your children(47)	1999	Alberta Health and wellness.	Canada	parents	Knowledge, Attitude, Skill
19	“It’s Easier Than You Think” Talking with your children about sexual and well-being(48)	2009	Published and distributed by sexual Health Access Alberta Funded by wild Rose Foundation and Community donors.	Canada	parents	Knowledge
20	Parent to Parent Guide on how To talk to children about sexuality(49)	2009	A Publication of Planned Parenthood Federation of America. Website: www.ppfastore.org	New York (USA)	parents	Knowledge, Attitude, Skill

## Discussion

Although valuable and effective packages and guidelines in relation to SE for children were found in this review, feasibility, and possibility of their usage in accordance with the Iranian culture was in question.

Although cultural influences may alter the efficiency of any given educational program, the majority of the included packages and guidelines agree on 1) parents’ role in SE; 2) education as the primary focus of SE; 3) parents as the primary sexuality educators; 4) attention to the values and culture of every society in SE for children; 5) effectiveness of the educational programs in children’s sexuality.

However, approaches to improving parents’ practical skills in SE for children were limited. In other words, parents’ competency in their children’s sexual behavior management in day-to-day practice was not the focus of attention in these programs.

Some packages and guidelines were designed for parents (Table 1). There is widespread agreement that parents are children’s first and foremost educators and that they play a central role in the development, growth, and management of children’s sexual behaviors (50–52).

This agreement can be found in several studies and guidelines such as Bersamin et al. (53); Vidourek (54); Goldman (55); Sexual Development and Behavior in Children Information for Parents and Caregivers (35), International Technical Guidance of Sexuality Volume II (36), Guidelines for Comprehensive Sexuality Education 3^RD^ editor kindergarten through 12^th^ Grade (38).

In addition to parents’ prominent role, some packages and guidelines were found targeting at teachers (Table 1). For instance, teachers’ role in children’s sexual health promotion is undeniable, and teachers can play an important role in promoting sexual health (56) and formal school-based education can help parents acquire’ the related skills and knowledge (28, 57–59).

Some packages and guidelines have been designed for parents and professionals (Table 1). Professionals work in the area of promotion of sexual health and education; in particular, curriculum and program planners, and educators in and out of a school setting, policy-makers, and health care practitioners. For parents who support the implementation of a comprehensive SE program, the quality of SE for their children will improve (60). Parents are given information and knowledge in this field by professionals in order to acquire the essential knowledge (61, 62) since parents does not have access to the appropriate resources (63, 64). Many resources pay attention to parents’ role as sexual educators, emphasize education of parents by professionals as the first sexual health promotion strategy for children (25, 44, 59), and focus on provision of educational materials for enhancing knowledge, so that adults can easily talk to their children (44, 65) and destroy barriers of negative attitude towards SE for children (60).

In educational packages and guidelines, attention to the values and culture of every society in SE for children has been emphasized, and this has been the strength of these programs. The influence of parents’ attitudes has extended well into all stages of life (53–55, 13). Sexuality is strongly influenced by family and community norms. As the sexual socialization of children, takes place first at home and then in the society, the role of culture and family values is very substantial and overshadows children’s sexuality (12, 21).

Many of these programs were related to developed countries where SE for children is widely recognized (25), yet it remains unacceptable in some countries, and Iran is not an exception. There is a lack of agreement on SE for children in Iran like other conservative societies. In developed countries, children receive formal and informal education by parents, school, and professionals; yet despite Iran’s progress in sexual and reproduction health and its movement toward healthy communities as defined by WHO (2004), a formal comprehensive SE for children does not exist (18, 66).

This study has some limitations as most of the packages and guidelines found in this review were stated theoretically. In other words, they were not based on experimental studies.

## Conclusion

The findings in this review emphasized the importance of SE for children. All programs focus on children’s sexual growth and development. Synthesis of the findings reveals that skill-building targeting parents are not practically specified throughout the studies, packages or guidelines. A possible explanation is that SE needs to be contextualized through a given society. Building skills for parents in management of their children’s’ sexuality must be the focus of SE programs. Children’ sexual development goals will be achieved if their first line educations become skillful.

General principles of these packages and guidelines have generalizability and usability for other countries such as Iran but some details of these packages and guidelines need to be repaired and modified according to the culture of each country. The implications of these findings for intervention design and development and further research are discussed.

## Ethical considerations

Ethical issues (Including plagiarism, informed consent, misconduct, data fabrication and/or falsification, double publication and/or submission, redundancy, etc.) have been completely observed by the authors.
